# CD4^+^CD25^+^FoxP3^+^ regulatory T cells and cytokines interact with estradiol in cases of missed abortion

**DOI:** 10.3892/etm.2013.1422

**Published:** 2013-11-25

**Authors:** WEIPING CAO, WENLIN XU, TINMEI CHEN, XIAOYING WANG, XINZHI WANG, JIAN QIU, NINTAO CHEN, YU MAO

**Affiliations:** 1 Department of Maternity and Child Health, Maternity and Child Health Hospital of Zhenjiang, Zhenjiang, Jiangsu, P.R. China; 2 Central Laboratory of Medicine, Maternity and Child Health Hospital of Zhenjiang, Zhenjiang, Jiangsu, P.R. China; 3 Department of Obstetrics, Maternity and Child Health Hospital of Zhenjiang, Zhenjiang, Jiangsu, P.R. China; 4 Jiangsu Center for Drug Screening, China Pharmaceutical University, Nanjing, Jiangsu, P.R. China; 5 School of Medicine, Jiangsu University, Zhenjiang, Jiangsu, P.R. China

**Keywords:** early missed abortion, CD4^+^CD25^+^FoxP3^+^ regulatory T cells, cytokine, interferon-γ, interleukin-4, estradiol

## Abstract

The aim of this study was to examine the interaction of estradiol (E2) with CD4^+^CD25^+^FoxP3^+^ regulatory T (Treg) cells and cytokines in cases of missed abortion (MA). The peripheral blood lymphocytes from patients with MA and controls (normal pregnancy and non-pregnant females) were isolated from the blood by Ficoll density gradient centrifugation. CD4^+^CD25^+^ Treg cells were isolated from peripheral blood mononuclear cells (PBMCs). The frequencies of CD4^+^CD25^+^FoxP3^+^ Treg cells and mRNA expression of transcription factor forkhead box protein 3 (FoxP3) in the peripheral blood of MA (n=33), normal pregnancy (n=33) and non-pregnant females (n=27) were determined by intracellular three-color flow cytometry and quantitative polymerase chain reaction (qPCR), respectively. The serum levels of interferon-γ (IFN-γ) and interleukin-4 (IL-4) were measured by enzyme-linked immunosorbent assay (ELISA) and a chemiluminescent immunoassay was used to examine the serum E2 levels. It was observed that the percentage of Foxp3^+^ T cells in the peripheral blood of patients with MA were lower compared with those in the normal pregnancy and healthy non-pregnant controls. The results demonstrated that MA patients exhibited decreased levels of a peripheral Th2-related cytokine (IL-4) and E2. Furthermore, the low levels of CD4^+^CD25^+^Foxp3^+^ T cells and IL-4 correlated positively with serum concentrations of E2. The data indicated that maternal immunological changes may reverse maternal tolerance in MA, and this phenomenon may be due to the interaction of E2 with CD4^+^CD25^+^Foxp3^+^ T cells and cytokines in MA.

## Introduction

Missed abortion (MA) is diagnosed when a pregnancy ceases to develop, but there is a delay in the expulsion of the products of conception. MA is a complication of early pregnancy that occurs in ≤15% of all clinically recognized pregnancies (1). Approximately 90% of all MAs occur prior to 14 weeks gestation, or in the first trimester. The etiology of MA is not fully understood. Although MA may occur due to chromosomal anomalies, hormonal problems, uterine abnormalities, infections and autoimmune disorders, in certain cases no cause is identified. Pregnancy is something of a physiological miracle in which an event that is normally forbidden, the propagation of foreign tissue, is accommodated for a defined period of time by the immune system. In order to achieve a successful pregnancy, the maternal immune system must be immunologically tolerant of the semi-allograft fetus. Incomplete tolerance may result in a disturbed pregnancy (2). Evidence suggests that CD4^+^CD25^+^ regulatory T (Treg) cells participate in the development of maternal tolerance to the fetus during pregnancy (3,4). CD4^+^CD25^+^ Treg cells are considered be crucial in peripheral tolerance, transplant tolerance and maternal tolerance to the fetus (5,6). The expression of forkhead box protein 3 (FoxP3) is regarded as characteristic of CD4^+^CD25^+^ Treg cells and differentiates them from activated CD4^+^ T cells (7). Normal pregnant patients exhibit an expansion of CD4^+^CD25^+^ Treg cells at the periphery compared with non-pregnant subjects. The percentage of CD4^+^CD25^+^ Treg cells has been observed to decrease to the levels of those in non-pregnant patients in the case of miscarriage (8). It seems that immune factors, such as decreased maternal tolerance, contribute to pathological pregnancy. Due to these observations, it has been proposed that Treg cells are crucial to maternal tolerance in humans.

The Th1 subset is defined by the specific production of interferon-γ (IFN-γ) and interleukin-2 (IL-2), and by the stimulation of cell-mediated immunity, whereas the Th2 subset specifically produces IL-4 and IL-10 which stimulate humoral immunity. Certain cytokines such as IL-4, IL-3 and IL-10 appear to be favorable to the success of pregnancy, whereas cytokines such as IL-2 and IFN-γ are reported to have deleterious effects (9). It has been proposed that a normal pregnancy is associated with a maternal predisposition to Th2-type immunity and that a preponderance to type 1 reactivity is associated with pregnancy failure. Numerous studies of unexplained recurrent spontaneous abortions (10), premature rupture of membranes and preterm delivery in humans have revealed a close association of these conditions with the increased production of certain type 1 cytokines by peripheral blood cells. The purpose of this study was to investigate whether the Th1/Th2 balance was broken in patients with MA.

Pregnancy induces substantial changes in hormone levels, initially in hormones produced by the *corpus luteum* and trophoblasts followed by complex alterations initiated by the hypothalamic-pituitary-adrenal (HPA) axis. E2 is a form of estrogen in the body derived almost entirely from the fetal-placental unit. Thus, maternal blood or urinary E2 is a good indicator of the health and well-being of the placenta and fetus. Estrogens have powerful effects on immune cells and regulate their proliferation, distribution and function (11). However, estrogen suppresses the maternal immune response in a manner that is poorly understood.

The pathogenesis of MA is complicated and multiple factors are involved in the formation of a clear clinical picture. We propose that the levels of E2 affect lymphocytes, such as Treg cells, in addition to the Th1/Th2 imbalance, which may be responsible for the pathogenic mechanism of development and progression of MA. To date, to the best of our knowledge, there have been no data regarding Treg cells and the effect of E2 on the immune system in patients with MA.

## Materials and methods

### Patients

In total, 33 MA patients with a median age of 28.4±5.71 years (range, 21–44 years) were included in this study. A first trimester MA was defined as ultrasound evidence of an intact gestational sac, no evidence of fetal cardiac activity [6 weeks from the last menstrual period (LMP)], a closed cervical os, and a history of no or minimal bleeding (12). The study group is referred to in the present study as the MA patient group. Patients with chromosomal anomalies, uterine abnormalities, infections and autoimmune disorders were not assigned to this group. The two control groups: one included 33 normal pregnant women in the first trimester and the other included 27 non-pregnant women. There were no significant differences in the age and pregnancy duration between the three groups (Table I).

This study has the approval of the Ethics Committees of the Maternity and Child Health Hospital (Zhenjiang, China). Written consent was obtained from all subjects following a full explanation of the procedure.

### Blood sample preparation

Venous blood ~8ml, was obtained by venipuncture from early MA (n=33) and healthy non-pregnant (n=27), and early-stage pregnancy patients (n=33). Of the 8 ml, 6 ml was heparinized for the isolation of peripheral blood mononuclear cells (PBMCs), while the remaining 2 ml was used for the preparation of serum. PBMCs were isolated for analysis by flow cytometry and quantitative polymerase chain reaction (qPCR) using Ficoll-Hypaque (Lymphoprep™; Nycomed Pharma, Oslo, Norway) density gradient centrifugation. Centrifugation was performed at 840 × g for 20 min at 20°C. The serum was separated from the specimens and stored at −70°C until required for cytokine determination using an enzyme-linked immunosorbent assay (ELISA) and a chemiluminescent immunoassay that was used to examine the serum levels of E2.

### Flow cytometry

To each tube, 100 μl prepared PBMCs (1×10^6^) were added, followed by 20 μl CD4/CD25 [fluorescein isothiocyanate/R-phycoerythrin (FITC/PE); eBioscience, San Diego, CA, USA]. The mixture was incubated in the dark for 30 min at 4°C and subsequently washed in cold flow cytometry staining buffer (BD Biosciences, San Jose, CA, USA). After decanting, the cell pellet was resuspended in the residual buffer and 1 ml freshly prepared fixation/permeabilization buffer (eBioscience), and incubated for a further 30–60 min in the dark at 4°C. The cells were washed with 2 ml permeabilization buffer followed by centrifugation and decanting of the supernatant. The cells were washed with 2 ml permeabilization buffer followed by centrifugation and decanting of the supernatant. The cells were blocked by adding 2 μl normal rat serum in ~100 μl cell suspension, for 15 min. Following the blocking step, without washing, 20 μl anti-human FoxP3 [PE-cyanine-5 (cy5); eBioscience] antibody was added and the cells were incubated at 4°C for at ≥30 min in the dark. The cells were washed with 2 ml permeabilization buffer (Cytoperm/Cytofix; Becton Dickinson, San Diego, CA, USA) followed by centrifugation (500 × g for 5 min at room temperature) and decanting of the supernatant; this was performed twice. Labeled cells were washed and analyzed by flow cytometry (Calibrate; Becton Dickinson, Palo Alto, CA, USA) using CellQuest software (Becton-Dickinson). In each case, staining was compared with that of the appropriately labeled isotype control antibody. The isotype control antibodies we used were Rat IgG2a Isotype Control FITC, Mouse IgG1 Isotype Control PE and Rat Kappa Isotype Contol PE-Cy5, respectively, and were purchased from eBioscience.

Flow cytometry was performed with a BD LSK flow cytometer (BD Biosciences). Data were collected and analyzed using CellQuest Pro software. Matched isotype controls were used to set quadrants and regions of positive staining. Cells were gated on lymphocytes using forward and side light-scattering properties and a minimum of 10,000 lymphocyte gated events were acquired. CD4^+^ T cell lymphocytes were analyzed with bivariate dot plots of CD25 vs. Foxp3. Treg cells are expressed as a percentage of CD4^+^CD25^+^FoxP3^+^ lymphocytes.

### RNA isolation and qPCR

Total RNA was extracted from individual PBMC preparations using TRIzol reagent (Invitrogen, Carlsbad, CA, USA) according to the manufacturer’s instructions. cDNA was prepared by reverse transcription with oligo(dT) (High-Capacity cDNA Reverse Transcription kit; Applied Biosystems, Foster city, CA, USA) from the total RNA extract. cDNA synthesis was performed using the High-Capacity cDNA Reverse Transcription kit (Applied Biosystems) according to the manufacturer’s instructions. Real-time PCR was performed in a 20 μl-system that contained 10 μl of 1X SsoFast EvaGreen Supermix (Bio-Rad, Hercules, CA, USA), 2 μl of cDNA, 6 μl of RNase/DNase-free water and 500 nM of each primer. The thermal cycler conditions were as follows: 30 sec at 95°C, followed by 40 cycles of 5 sec at 95°C and 10 sec at 60°C. A melting curve analysis was performed for each reaction with a 65–95°C ramp. The threshold cycle at which the fluorescent signal reached an arbitrarily set threshold near the middle of the log-linear phase of the amplification for each reaction was calculated, and the relative quantity of mRNA were determined. The mRNA levels were normalized against the mRNA levels of the housekeeping gene, β-actin. qPCR for FoxP3 and a reference gene (β-actin) was performed in a Lightcycler Instrument (Roche Molecular Diagnostics, Mannheim, Germany) with the SYBR-Green Mastermix kit (Takara, Shiga, Japan). FoxP3 expression data was subsequently normalized relative to β-actin. The primer sequences for qPCR are shown in Table II.

### Cytokine measurement using ELISA

Serum IL-4 and IFN-γ concentrations were measured by commercial ELISA according to the manufacturer’s instructions (Bender MedSystems, Burlingame, CA, USA). All samples were analyzed in duplicate.

### E2 levels

E2 was quantified in the serum of subjects from MA patients and control groups by chemiluminescence immunoassay (Architect-i2000; Abbott Laboratories, Green Lakes, IL, USA). The absorbance value was monitored at 450 nm on a microplate reader (Safire 2, Tecan, Switzerland).

### Statistical analysis

Statistical analysis was performed with GraphPad Prism version 4.0 (GraphPad software Inc., San Diego, CA, USA). Data are presented as the means ±SD. P<0.05 was considered to indicate a statistically significant difference. As determined by one-way analysis of variance (ANOVA) or the Student’s t-test. Pearson’s correlation was used to analyze correlations between the levels of estradiol, Treg cells and IL-4 in MA patients.

## Results

### Levels of FoxP3-expressing CD4^+^CD25^+^ T cells in MA patients as determined by flow cytometry with intracellular staining

**Figure 1:**
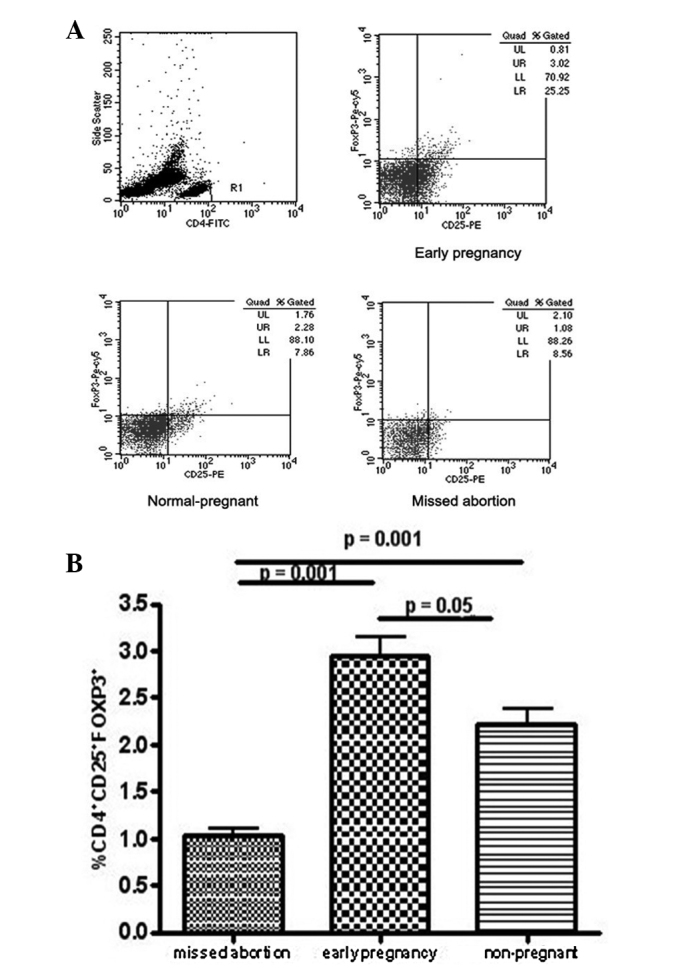
Flow cytometric analysis of the expression of the transcription factor forkhead box protein 3 (FoxP3). (A) Three color analysis of expression of FoxP3 in cells stained for CD4 [fluorescein isothiocyanate (FITC)], CD25 [R-phycoerythrin (PE)] and FoxP3 [PE-cyanine-5(Cy5)] on freshly isolated Treg cells. Plot for FoxP3 vs. CD25 expression of gated CD4^+^ T cells. (B) FoxP3-expressing cells of the peripheral blood in missed abortion were fewer than those in normal pregnancy and non-pregnant subjects.

Immunostaining for FoxP3 was conducted in order to detect CD4^+^CD25^+^ T cells, the most reliable markers for CD4^+^CD25^+^ Treg cells. The flow cytometry results demonstrated that the levels of FoxP3^+^ cells in the peripheral blood of MA patients were lower than those in normal pregnancy and non-pregnant subjects, suggesting that Treg cell levels were reduced in the peripheral blood during MA (Fig. 1).

### FoxP3 mRNA expression levels in the peripheral blood of MA patients as determined by qPCR

**Figure 2:**
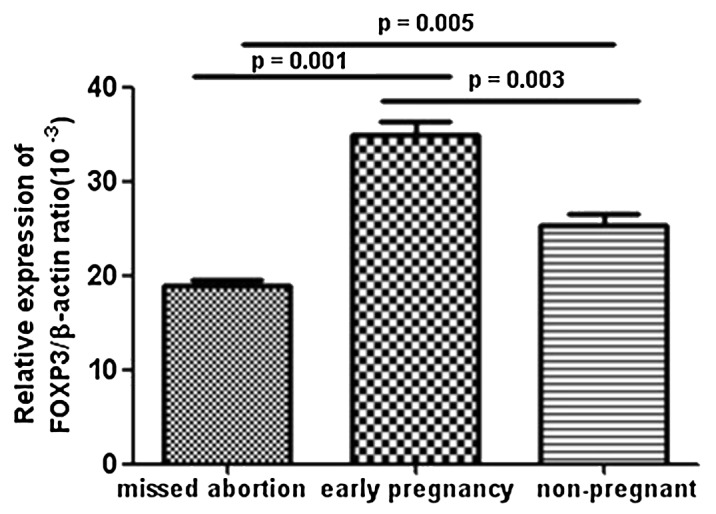
Reduction of forkhead box protein 3 (FoxP3) mRNA expression in the peripheral blood of patients with missed abortion compared with that in early pregnancy and healthy non-pregnancy patients.

In order to confirm the previous observations, the levels of the transcription factor specific for T-cell subsets were determined by qPCR. As shown in Fig. 2, decreased mRNA expression levels of the Treg cell-specific transcription factor, FoxP3, were observed in patients with MA compared with those in normal pregnancy and healthy non-pregnant females. These results were consistent with the flow cytometric analysis of Treg cells.

### Serum cytokine concentrations as determined by ELISA

**Figure 3:**
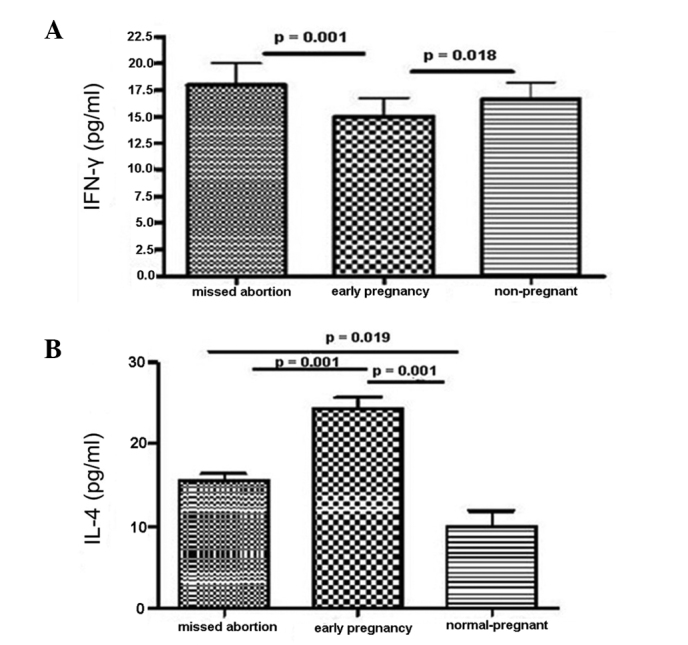
Serum cytokine concentrations were determined from the peripheral blood samples obtained from missed abortion patients and normal pregnancy and non-pregnant females. The concentrations of serum (A) interferon-γ (IFN-γ) and (B) interleukin-4 (IL-4) were examined by enzyme-linked immunosorbent assay (ELISA) using specific cytokine detection kits.

The intracellular expressions of IL-4 and IFN-γ were determined in the serum by ELISA as an indicator of cytokine production. As shown in Fig. 3, the IFN-γ expression levels in MA patients were higher compared with those in the control groups (Fig. 3A). By contrast, the MA patients demonstrated lower production levels of the Th2 cytokine IL-4 than those in the control groups (Fig. 3B). These data suggest an abnormal immune response in MA patients, characteristic of a shift to Th1-type immunity.

### Correlation of E2 with Treg cells and IL-4 in MA patients

**Figure 4:**
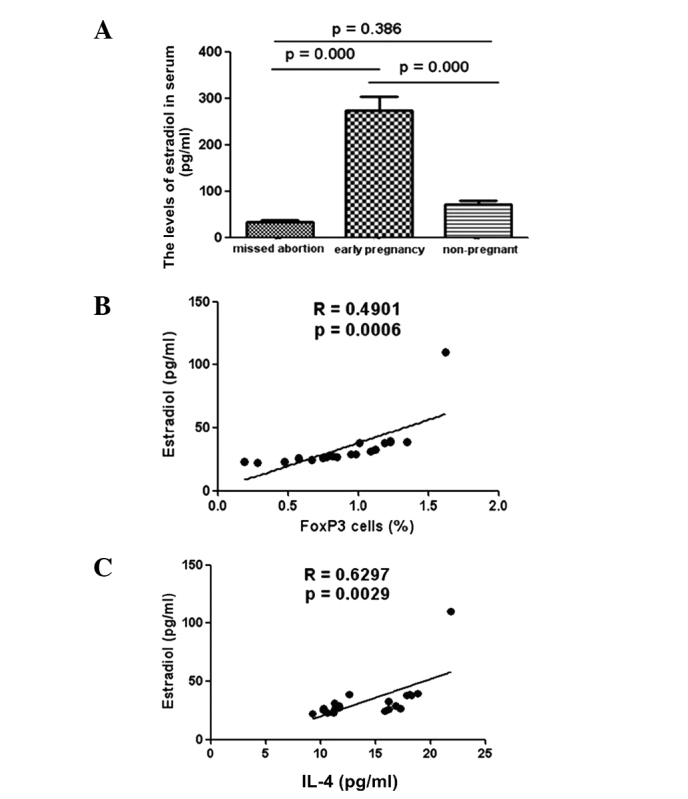
(A) Alteration of estradiol level in the serum of missed abortion patients. (B) Correlation between the levels of estradiol and the percentage of forkhead box protein 3 (FoxP3)^+^ cells. (C) Correlation between the levels of estradiol and interleukin-4 (IL-4) concentrations in missed abortion patients.

To explore whether E2 expression levels were affected during the pathogenesis of MA in humans, the serum E2 levels of patients were examined. Lower levels of E2 were detected in the serum samples of MA patients than in the samples from healthy pregnant subjects (Fig. 4A). Further analysis revealed a positive correlation between the low levels of E2 and reductions in the production of Treg cells in MA patients (Fig. 4B). Furthermore, low levels of E2 expression in MA patients correlated positively with the IL-4 levels of these patients (Fig. 4C).

## Discussion

The fetus has been viewed as a semi-allograft to the maternal host. However, pregnant patients do not usually experience fetus rejection. The acceptance of the semi-allogeneic fetus within the maternal environment requires mechanisms of tolerance. Evidence has suggested that CD4^+^CD25^+^ Treg cells participate in the development of maternal tolerance to the fetus during pregnancy (13). It has been confirmed that an augmentation in the quantity of Treg cells during pregnancy and, most importantly, diminished numbers of Treg cells, are associated with immunological rejection of the fetus (14). In the present study, a difference in the frequency of CD4^+^CD25^+^ Treg cells in the peripheral blood of MA patients and normal early pregnancy and non-pregnant subjects was demonstrated; normal pregnant patients demonstrated an expansion of CD4^+^CD25^+^ cells at the periphery compared with non-pregnant subjects. Furthermore, significantly lower frequencies of Treg were found in MA patients. Analyses have suggested that decreasing levels of Treg cells indicate that they have a role in maternal alloantigen tolerance during pregnancy (15). In humans, low levels of circulating CD4^+^CD25^+^ Treg cells have been identified to be predictive of a risk of miscarriage in newly pregnant patients with a history of failure, suggesting that the level of peripheral Treg cells may serve as a superior pregnancy marker (16,17). The findings of the present study imply that CD4^+^CD25^+^ Treg cells may also play a pivotal role in MA. The present study is, to the best of our knowledge, the first to show that the frequency of CD4^+^CD25^+^FoxP3^+^ Treg cells in peripheral lymphocytes is lower in patients with MA than in controls.

The balance of CD4^+^Th1 and Th2 cells plays a role in the pathogenesis of MA patients (18). Based on the analysis of IFN-γ and IL-4 production, a dominance of Th1 cell activity over Th2 cell activity has been observed in MA patients. In the present study, IFN-γ expression levels in MA patients were significantly higher than those in the control group, whereas the MA patients exhibited lower levels of the Th2 cytokine, IL-4. These data suggest an abnormal immune response in MA patients with a characteristic shift to Th1-type immunity (10).

Sex hormones, such as E2, play a vital and complex role during pregnancy and interact with each other to mediate various parts of the pregnancy process. The majority of the E2 in adult females during reproductive years is derived from the ovaries, while E2 levels increase markedly only during pregnancy. During pregnancy E2 is produced by the mother, placenta and fetus. Furthermore, the blood levels of these hormones change over the course of pregnancy; in certain cases they correlate with changes in the maternal immune response (19). In the present study, E2 levels were identified to be lower in the MA group compared with those in the normal pregnancy group, while no clear difference was observed between MA patients and healthy non-pregnant patients. However, further analysis revealed a positive correlation between low levels of E2 and decreased levels of Treg cells and IL-4 production in MA patients. These results indicated that decreased E2 levels may promote a shift in the Th1/Th2 balance toward Th1 dominant immunity, leading to low levels of IL-4. Furthermore, E2 may be one of the regulators of Treg cell proliferation and differentiation (20,21). The phenomenon illustrates the intricate working of hormone-immune system interaction in MA. However, the exact mechanism of action for E2 in various cell types of the immune system with MA remains unclear.

In conclusion, this study detected low levels of E2 in the sera of MA patients, which correlated with decreased peripheral blood levels of Treg cells and IL-4 in MA patients. Thus, these results have revealed the previously unappreciated association of E2 with Treg cells and cytokines in the pathogenesis of MA. The findings, which show a positive correlation between low levels of E2 expression and decreased Treg cell populations and IL-4 levels in MA patients, may facilitate the potential development of novel therapies targeting the hormone-immune system pathway for the treatment of human MA.

## Figures and Tables

**Table I:** Characteristics of missed abortion patients and control groups in the study.

Groups	N	Maternal age (years)	Gestational age (days)
Missed abortion	33	28.4±5.71	52.75±1.96
Normal pregnancy	33	28.5±5.10	52.35±1.63
Non-pregnant subjects	27	27.0±5.67	

Missed abortion vs. control groups, P=0.89; missed abortion vs. normal pregnancy, P=0.16.

**Table II:** Primers for qRT-PCR.

Gene	Forward primer	Reverse primer
β-actin	5′-TTCTGTCAGTCCACTTCACCA-3′	5′-CCAGCAGGTCTGAGGCTTG-3′
FoxP3	5′-TGAGAAGGACAGGGAGCCAA-3′	5′-GAGAAGCTGAGTGCCATGCA-3′

qRT-PCR, quantitative reverse transcription polymerase chain reaction; FoxP3, forkhead box protein 3.

## Abbreviations

MA: missed abortion
PBMCs: peripheral blood mononuclear cells
IFN-γ: interferon-γ
IL-4: interleukin-4
E2: estradiol
Treg cells: CD4^+^CD25^+^FoxP3^+^ regulatory T cells
ELISA: enzyme-linked immunosorbent assay
